# Effects of the HEP® (Homeostasis–Enrichment–Plasticity) Approach in preterm infants with increased developmental risk: a randomized controlled study

**DOI:** 10.3389/fped.2025.1606490

**Published:** 2025-09-25

**Authors:** Gamze Cagla Sirma, Ayse Zengin Alpozgen, Aymen Balikci

**Affiliations:** ^1^ Department of Physiotherapy and Rehabilitation, Institute of Graduate Studies, Istanbul University-Cerrahpaşa, Istanbul, Türkiye; ^2^ Department of Occupational Therapy, Fenerbahce University, Istanbul, Türkiye; ^3^ Division of Physiotherapy and Rehabilitation, Faculty of Health Sciences, Istanbul University-Cerrahpaşa, Istanbul, Türkiye; ^4^ Sense on Ltd., Istanbul, Türkiye

**Keywords:** early intervention, environmental enrichment, development, prematurity, infants at risk

## Abstract

**Background and objectives:**

This study investigated the effectiveness of the Homeostasis-Enrichment-Plasticity (HEP) Approach in preterm infants with increased developmental risk, compared to the Traditional Treatment (TT) intervention for physical and occupational therapy.

**Materials and methods:**

Twenty-nine preterm infants (adjusted age, 4–10 months) were randomly assigned to two groups: the HEP Approach group and the TT group. The Peabody Developmental Motor Scales-2 (PDMS-2), Test of Sensory Functions in Infants (TSFI), and Beck Anxiety Inventory (BAI) were administered pre- and post-intervention. The intervention was implemented weekly for 12 weeks.

**Results:**

The baseline characteristics of the infants were similar. At the end of the treatment, a significant time effect was observed in motor skills and sensory functions across both groups, with improvements in all PDMS-2 and TSFI subtests (*p* < 0.05). Significant time × group interactions showed greater improvements in the HEP Approach group compared to the TT group for Fine Motor Quotient: *F* = 10.818, *p* = 0.003; Gross Motor Quotient: *F* = 5.691, *p* = 0.024; and Total Motor Quotient: *F* = 21.109, *p* < 0.001. For TSFI, the HEP Approach group showed greater improvements in Adaptive Motor Functions (*F* = 13.794, *p* = 0.001), Visual-Tactile Integration (*F* = 7.410, *p* = 0.011), and Total score (*F* = 11.316, *p* = 0.002). No significant time*group interactions were found for Reactivity to Tactile Deep Pressure, Ocular Motor Control, and Reactivity to Vestibular Stimulation (*p* > 0.05). Parental anxiety, measured by BAI, decreased significantly in both groups (*F* = 8.72, *p* = 0.006), but no significant time × group interaction was found (*p* > 0.05), indicating similar reductions in both groups.

**Conclusion:**

The HEP Approach demonstrated superior outcomes compared to the TT intervention in improving motor skills and sensory functions in preterm infants, while both interventions reduced caregiver anxiety.

## 1 Introduction

Globally, 1 in 10 infants is born preterm (<37 weeks gestation). Preterm birth rates have remained relatively stable over the past decade, although they have increased in some regions. In 2020, it was estimated that approximately 1 million newborns would die as a result of preterm birth problems, and millions more would live with disabilities that affect them and their families for the rest of their lives (1).

Preterm infants often spend their early weeks or months in the Neonatal Intensive Care Unit (NICU), an environment vastly different from the intrauterine milieu. NICUs expose infants to intense lighting, excessive noise, and painful procedures, all of which can disrupt brain development, neural network connectivity, and physiological responses (2). Consequently, preterm infants face significant risks in sensory, motor, emotional, cognitive, and social development, particularly those born before 34 weeks of gestation (3–5). Moreover, preterm birth increases stress, anxiety, and depression levels in families and imposes considerable medical and socioeconomic burdens on society.

Early intervention (EI) is crucial in supporting the development of preterm infants who face developmental risks (6). EI is defined as services provided to enhance brain connections during critical periods of an infant's central nervous system development, particularly from birth to 3 years of age (6, 7). For decades, EI has been implemented worldwide to improve long-term positive outcomes in infants' development and family well-being. However, the literature has not consistently reported the effectiveness of EIs in improving infant development (8). Possible reasons for this discrepancy may include the type of intervention, inadequate intervention dosage, timing of intervention initiation, and the efficacy of the intervention.

One of the types of EI for gross and fine motor development provided by physical and/or occupational therapists is traditional treatment (TT), which is commonly recognized worldwide as being based on neurodevelopmental therapeutic principles, and focusing on balance and correction reactions, weight-bearing, and postural control (9, 10). However, new intervention approaches are required because TT is traditionally a “hands-on” method where the physio or occupational therapist engages directly with the child, manipulates their position, and plays the role of a teacher in the relationship with the infant and caregiver. In this approach, active and continuous engagement on the part of the child remains limited because such stimuli can only be provided during therapy sessions in the clinic and through recommendations given to families for home practice.

Recent models of EI have been increasingly shaped by insights derived from enriched environment (EE) research and ecological theories of development (11–16). Findings from EE studies, particularly those conducted in animal models, have consistently demonstrated that systematic environmental enrichment can exert profound influences across multiple domains, including developmental trajectories, behavioral adaptation, physiological regulation, neuroanatomical organization, and even molecular processes (17–20). In animal research, despite variations in specific methods and emphases, enrichment paradigms have relied on a common set of ten principles—physiological homeostasis, safety, multisensory stimulation, spatial characteristics of the environment, novelty, challenge, enjoyment, sustained engagement, social opportunities, and active exploration—as the primary dimensions through which enrichment is operationalized) (21, 22). Building on this evidence base, recent EI approaches selectively integrate different combinations of these principles while also drawing upon diverse ecological frameworks of human development. This dual grounding enables programs to establish a shared conceptual foundation, yet the particular constellations of principles and theoretical orientations they adopt vary considerably. Such variability reflects distinct emphases in how developmental processes are conceptualized, how experiential contexts are organized, and how implementation strategies are prioritized in practice (22–24).

EE-based EI approaches aim to enhance neurodevelopment by increasing multisensory stimulation, opportunities for physical activity, and social engagement, primarily through the encouragement of spontaneous exploratory behaviors (22, 23, 25). Contemporary programs such as GAME and START-Play exemplify evidence-based applications of ecological principles. The GAME program, grounded in motor learning and Dynamic Systems Theory (DST), emphasizes goal-oriented, intensive motor training delivered in the home environment. Families collaborate with therapists to set individualized developmental goals that encompass both motor development and broader health-related factors such as sleep and nutrition. Motor tasks are scaffolded to encourage active, self-initiated movements, while parents are coached to observe, support, and problem-solve, thus integrating variability and progressive challenge within structured home programs (14). Similarly, START-Play, informed by principles of Embodied Cognition, is delivered in the infant's natural environment and combines motor challenges with early cognitive constructs. Caregivers provide social support, scaffold learning, and facilitate active engagement in “just-right” challenges (13). Viewed through the lens of EE principles, both interventions incorporate multiple domains such as active exploration, social opportunities, and sustained engagement; however, they assign comparatively less emphasis to aspects like homeostasis, safety, spatial features of the environment, and certain dimensions of multisensory stimulation. Importantly, even when grounded in similar EE principles, the two approaches interpret, prioritize, and operationalize these principles differently, reflecting the distinct theoretical perspectives from which they emerge.

The HEP Approach, developed by Balıkcı, builds on the foundations of EE by offering a comprehensive framework that systematically incorporates all ten core principles (22). It is designed as a clinic-based intervention and parent coaching model which is provided with parallel parent-directed activity implementation in the home contexts; and therapist monitoring of infants' natural environments through online follow-up. Provision of the intervention initially in the clinic provides several advantages: infants are exposed to an already EE with diverse sensory and motor opportunities, while parents receive structured coaching in a supportive setting. Following the clinic sessions, families transfer and adapt the coached strategies into their daily routines and natural contexts, supported by online follow-up, which serves to guide, reinforce, and sustain this integration in the home environment. The HEP Approach is grounded in multiple theoretical perspectives, including Gibson's Ecological Perception Theory, Perception-Action Theory, Sensory Integration, and the Person–Environment–Occupation (PEO) Model, Dynamic Systems Theory (DST), Neuronal Group Selection Theory (22–24). In line with evidence supporting family-centered, goal-oriented, and coaching-based practices in pediatric occupational therapy (26–28), the HEP Approach empowers caregivers through structured yet flexible strategies that foster co-regulation, sensory–motor development, and environmental adaptation. In practice, homeostasis is prioritized, with Dynamic Systems and Sensory Integration theories suggesting that regulation in this domain can facilitate progress across other developmental areas. Families are guided in strategies such as rhythmic rocking, adjusting voice tone, or narrating daily events to enhance self-regulation. From the perspectives of Sensory Integration and Perception–Action theories, this approach underscores the importance of leveraging a child's strongest sensory systems for learning and development, supported by targeted environmental modifications—for instance, positioning infants with strong visual or auditory abilities in upright postures to enhance engagement. For infants at increased risk, delayed motor development may restrict opportunities for independent floor-based exploration. By facilitating upright positioning, caregivers not only enhance infants' sense of security through greater environmental control but also enable active tactile and auditory exploration with the hands which can address developmental sensory and motor challenges. In this way, structured environmental adaptations and carefully designed activity opportunities create enriched contexts for discovery that simultaneously support sensory processing, praxis, and broader developmental outcomes.

Based on the PEO model and Sensory Integration theory, spatial features of the environment and the objects therein are organized to provide safe, “just-right” challenges, such as placing a non-sitting infant in a supportive basket to encourage exploration with hands and eyes. In addition, activities and objects are analyzed for sensory features and praxis challenges to provide just-right and adaptive opportunities for interaction. Similarly, consistent with Perception–Action principles, activities and environments are structured to maximize opportunities for active exploration and self-initiated movement, for instance, through the use of baby walkers or bouncers to promote mobility in infants with restricted motor control. By integrating these theoretical perspectives with the core principles of EE into a unified and ecologically valid framework, the HEP Approach introduces a distinctive combination of practices to the field of early intervention. Supplementary Table S1 provides a point-by-point comparison of these three interventions to illustrate the differences and similarities of the approaches (Supplementary Table S1).

This study hypothesizes that the HEP Approach will demonstrate greater effectiveness in improving motor skills and sensory functions of preterm infants with increased developmental risk and a corrected age between 4 and 10 months compared to TT. Furthermore, it posits that the HEP Approach will better address caregiver anxiety by fostering active parental involvement in the intervention process.

## 2 Materials and methods

### 2.1 Study design

Following CONSORT guidelines, a prospective, randomized, controlled study was conducted between January 2022 and January 2023 at Istanbul University-Cerrahpaşa, with both outcome assessors and statistical analysts blinded to group allocation. The protocol for this study was approved by the Research Ethics Committee of Istanbul University-Cerrahpaşa (2022/09) and implemented per the Declaration of Helsinki Ethical Principles. All parents signed a written informed consent form before enrollment. The study protocol was also prospectively registered at ClinicalTrials.gov (NCT05261503).

### 2.2 Sample size determination

The sample size of the study was calculated using the G*Power 3.1 program. The sample size calculation was based on the mean and standard deviation values for the Peabody Developmental Motor Scales 2-Total Motor Quotient from a similar study in the literature (29). The study was designed with a power of 80%, a margin of error of 5%, and an effect size of 1.01 (*df* = 12; *t* = 1.782). The sample size was calculated to be 26 participants, with 13 participants in each group. The dropout rate was set at approximately 20%, considering the three-month intervention period, during which challenges such as loss to follow-up, scheduling conflicts, or unforeseen circumstances might arise. Based on this assumption, the sample size was determined to be 32 participants to ensure sufficient data for analysis.

### 2.3 Participants

Thirty-two participants were recruited from three local hospitals in Istanbul, Türkiye: two private and one public. Following the neonatologists' referral, a physiotherapist conducted preliminary telephone and in-person interviews with the families to determine if they met the inclusion criteria.

To assure a homogeneous population for this study, infants with corrected ages between 4 and 10 months and gestational ages less than 34 weeks, without any systemic illness or congenital anomaly, whose families agreed to participate in the study, and who had access to the WhatsApp application, were included in the study. Infants who had intraventricular haemorrhage (Grade III and IV), had significant vision or hearing problems, febrile seizures, medical conditions that hindered active participation in the study, or participated in other experimental rehabilitation studies were excluded as they potentially presented excessive diversity of cognitive and motor abilities in the sample for this current study.

**Figure 1:**
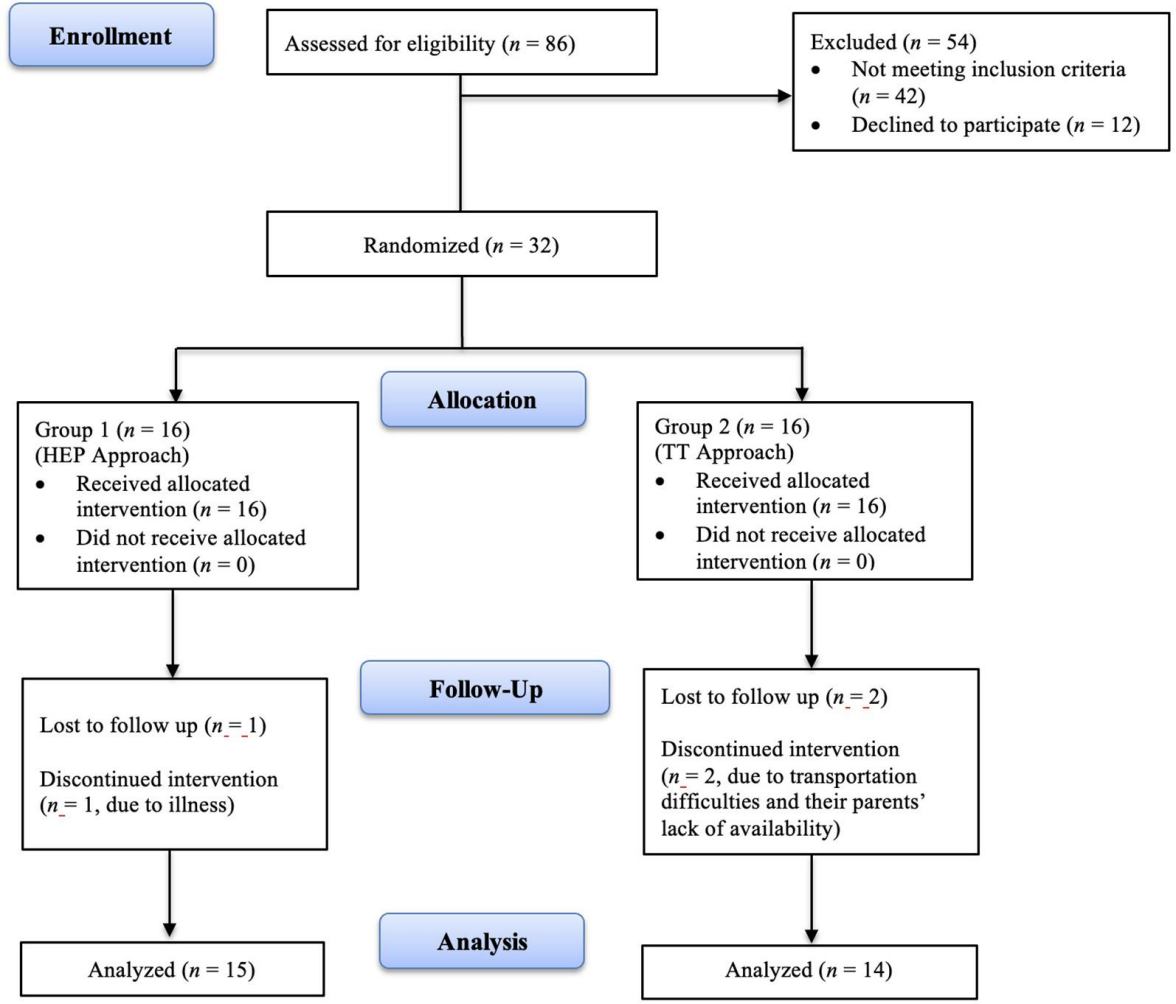
Flow chart of participant and outcome assessment through the trial.

Participants who met the inclusion criteria were randomly assigned to two groups. Stratified randomization was conducted by an independent researcher who was not involved in recruitment, intervention, or outcome assessment, to ensure allocation concealment. The gestational age and corrected age of the participants were used as criteria for stratified randomization. According to these criteria, eight blocks (assigned numbers 1–8) were created with four individuals in each block. Equal allocation through block randomization was achieved using www.sealedenvelope.com, a centralized web-based service for clinical trials. The participants were divided into two equivalent groups labeled as Group 1 (*n* = 16) and Group 2 (*n* = 16) to ensure blinding during group assignment. Figure 1 illustrates participants' flow and retention through the study.

### 2.4 Measures

Before and after the intervention, a series of assessments were collected through detailed parent interviews and systematic observation of behaviour in the clinical setting. Assessments included the Peabody Developmental Motor Scale-2 (PDMS-2), the Test of Sensory Functions in Infants (TSFI), and the Beck Anxiety Inventory (BAI). A physiotherapist with over five years of experience in pediatrics, blinded to the intervention groups, conducted the assessments.

#### 2.4.1 Peabody Developmental Motor Scales—Second Edition (PDMS-2)

The PDMS-2 consists of six subtests that assess children's gross and fine motor skills from 0 to 5 years of age. Each of the PDMS-2 subtests contributes to the calculation of the Total Motor Quotient (TMQ). For infants between 0 and 12 months, reflexes, stationary, and locomotion subtests contribute to the Gross Motor Quotient (GMQ) score, while grasping and visual motor integration subtests contribute to the Fine Motor Quotient (FMQ) score. According to the manual, standard scores indicate the following: 17–20 “very superior,” 15–16 “superior,” 13–14 “above average,” 8–12 “average,” 6–7 “below average,” 4–5 “poor,” 1–3 “very poor” performance. The test is completed in about 45–60 min, but may take less time for infants (30). The PDMS-2 is recognized as a reliable and valid tool for assessing motor development of premature infants. A culturally adapted version of the measure was used for test administration (31).

#### 2.4.2 Test of Sensory Functions in Infants (TSFI)

The TSFI is a therapist-administered standard performance test of sensory functioning for infants between the ages of 4 and 18 months. The test consists of 5 subsections and 24 items. The subsections of the test are reactivity to tactile deep pressure (RTDP), adaptive motor functions (AMF), visual-tactile integration (VTI), ocular-motor control (OMC), and reactivity to vestibular stimulation (RVS). The test requires the infant to be stimulated and interact with various materials, and the clinician observes and scores the infant's responses. The total score ranges from 0 to 49, with higher scores indicating better sensory processing. In the standard distribution curve, scores above −1 SD are scored as “normal,” scores between −1 SD and −2 SD are scored as “at-risk,” and scores below −2 SD are scored as “deficient” (32). The validity and reliability study of the adapted version of the test was conducted in 2014. The Cronbach Alpha coefficient of the scale was calculated as 0.875 (33).

#### 2.4.3 Beck Anxiety Inventory (BAI)

The BAI includes 21 emotional and somatic anxiety symptoms rated on a 4-point scale according to the severity experienced. The total score ranges from 0 to 63: 0–7 represent minimal, clinically insignificant symptoms; 8–15 represent mild anxiety symptoms; 16–25 represent moderate symptoms; and scores between 26 and 63 represent severe symptoms (34). The nationally adapted version of the inventory is valid and reliable, with a Cronbach's alpha of 0.94 (35).

### 2.5 Intervention

#### 2.5.1 HEP Approach

**Figure 2:**
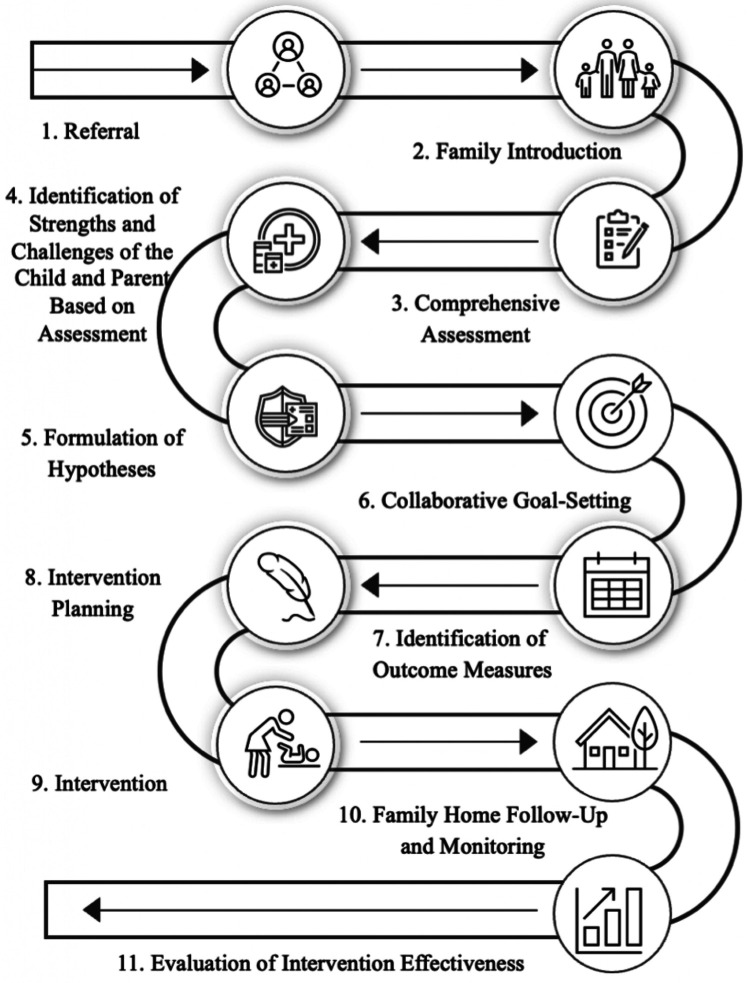
Illustration of the 11 phases of the HEP Approach.

The HEP Approach is a safe, feasible, and acceptable form of intervention for clinical implementation and participation by most parents of premature infants (20). In our study, the intervention process adhered to a well-structured 11-phase model which has been piloted and found to be effective (Figure 2) (24). In phase one, participants were directly referred to the program. Phase two involved a family orientation session to introduce them to the HEP Approach. In phase three, a comprehensive assessment of both the child and family systems was conducted. Phase four focused on identifying the strengths and challenges of the family and infant, based on the evaluation. In phase five, hypotheses were developed regarding the influence of underlying variables or systems on the child's domains of difficulty. During phases six and seven, families engaged in collaborative goal formulation and identified desired outcomes. Phase eight was dedicated to intervention planning. In phase nine, the intervention was implemented using a personalized approach, which typically includes four stages: promoting self-regulation and homeostasis in the child, adjusting the physical and social home environment to support success, expanding and diversifying interactions with the environment, and fostering family independence and autonomy in creating supportive environments for the child. Phase ten involved monitoring and follow-up with families, and phase eleven assessed the achievement and progress of the expected goals.

**Figure 3:**
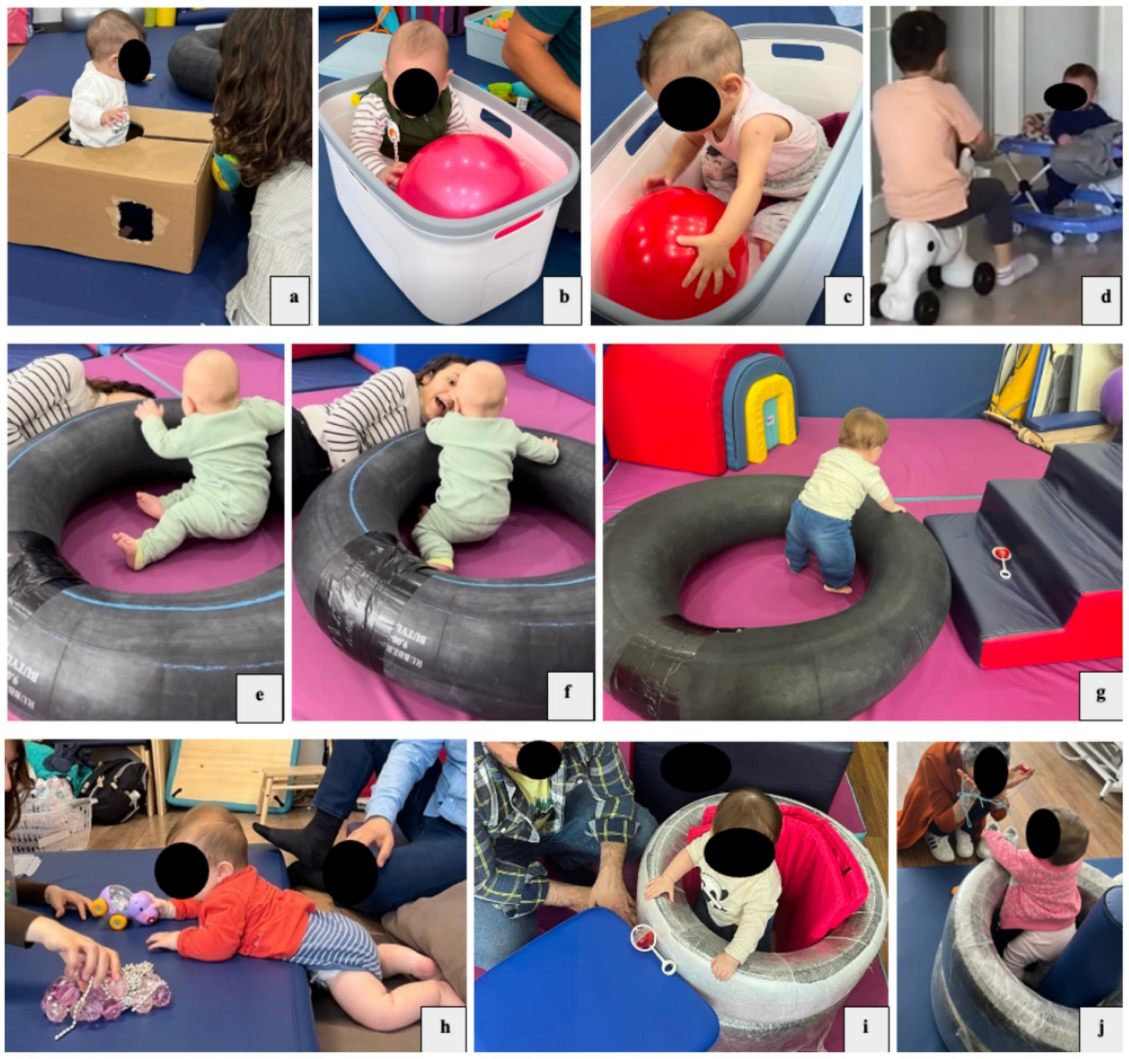
HEP approach intervention. **(a)** The infant is enclosed in a cardboard box, interacts with objects or the therapist/family, and explores a variety of possible actions with his/her body in a sitting position. The box provides a safe environment for exploring different movement possibilities, such as moving from sitting to upright; **(b,c)** the infant, who cannot sit unsupported, explores opportunities for interaction and movement in the seating area organized with a laundry basket; **(d)** the infant has opportunities to explore the environment (different parts of the room or different rooms) and different movement possibilities in his/her body with the baby walker. It also motivates the infant to interact with siblings and other family members; **(e–g)** an inner tube is used for the baby to explore different movement possibilities in the sitting position; **(h)** arrangements are made for the baby to explore the floor with his/her hands and feet in the prone position and to explore opportunities to interact with objects and people with his/her eyes and ears; **(i,j)** a safe space of “just right” dimensions is created for the baby to stand in. Explores different movement possibilities in the standing position through opportunities to interact with objects and people.

A physiotherapist, specializing in pediatric rehabilitation and trained by the developer of the HEP Approach (third author), provided the HEP intervention to participants in weekly 45-min sessions over a 12-week period in line with the 10 core elements of the approach (Supplementary Table S2). The frequency and duration of the intervention has been shown to be sufficient to result in positive changes in previous studies (22–24). HEP Approach intervention examples are presented in Figure 3. When necessary, the intervention was conducted under the supervision of the author of the HEP Approach, with the participant's identity anonymized, and the processes were monitored through inquiries. The clinic-based coaching sessions of the HEP intervention took place in a 40-square-meter room, where both the parent and child participated. The room was equipped with various standard sensory-motor therapeutic materials, including gym balls, foam blocks, barrels, rollers, a ball pit, mats, and swings. The initial provision of the intervention in the clinic offered important advantages: infants engaged in a structured, enriched environment with varied sensory–motor opportunities, while parents received guided coaching in a supportive context. This arrangement facilitated both active child participation and parental skill acquisition before strategies were transferred to the home setting. All clinic-based sessions were video-recorded, and parents were coached on effective interaction strategies with their children and on creating a supportive home environment. The parents implemented the home-based portion of the HEP Approach by applying the strategies learned in the clinic at home. Families provided weekly videos to the therapist via WhatsApp, who then offered constructive feedback, additional coaching and encouragement to the caregivers.

#### 2.5.2 Traditional treatment (TT)

The infants in the TT group were referred to physiotherapy and continued their routine medical examinations. Expert pediatric physiotherapists with Bobath certification led the infants in this group's sessions. Information about session attendance and intervention contents was obtained from the participants' files. All participants completed 12 weeks of 45-min sessions. The interventions were carried out by selecting activities that targeted intervention goals from among practices supporting proximal stabilization, weight transfer, balance, and postural control in prone, supine, sitting, and standing positions, corresponding to the developmental stages of infants. The activities performed during the session were taught to the caregivers at the end of the session and given as homework to integrate them into their daily lives. Caregivers received weekly reminders via WhatsApp to complete the assigned home activities. Moreover, video-based feedback and follow-up procedures—also conducted via WhatsApp—were instituted to enhance caregiver compliance and ensure the continuity of the intervention within the home setting. Examples of the TT intervention and its goals applied to these infants are provided in Supplementary Table S3. In addition, a timeline-based comparison of the HEP Approach and traditional treatment is presented in Supplementary Table S4.

### 2.6 Statistical analysis

All data were analyzed using the Statistical Package for the Social Sciences (SPSS, Version 26; SPSS Inc., Chicago, IL, USA). The kurtosis and skewness values of the variables, ranging between +1.5 and −1.5 verified the normality of the distribution (36, 37). Since all data were normally distributed, parametric tests were applied. Categorical variables were compared using Fisher's exact test and the chi-squared test. A 2 × 2 Repeated Measures Analysis of Variance (rANOVA) was conducted to evaluate pre- and post-treatment outcomes and the interaction effects of the treatment programs across groups. Results, including F and *p* values as well as partial eta squared (*P_η_*^2^) values, which indicate effect sizes, are summarized in the tables. Effect sizes were interpreted as follows: values between 0.01 and 0.06 indicate a small effect size, values between 0.06 and 0.14 indicate a medium effect size, and values greater than 0.14 indicate a large effect size.

## 3 Results

### 3.1 Participants

Thirty-two infants were recruited and randomized into the HEP Approach group (*n* = 16) and the TT group (*n* = 16). Following the interventions, data from 29 infants (*n* = 15 in the HEP Approach; *n* = 14 in the TT) were analyzed, with a dropout rate of 9.375%. The flow of participants is presented in Figure 1. The mean corrected age of the infants was 25.13 weeks (SD = 7.99) in the HEP Approach group and 24.14 weeks (SD = 8.63) in the TT group. The mean gestational age was 28.93 weeks (SD = 2.81) in the HEP Approach group and 29.79 weeks (SD = 3.53) in the TT group. Among the participants, 18 were male (62.1%) and 11 were female (37.9%). Sociodemographic and baseline characteristics of the infants and their parents are summarized in Table 1. No statistically significant differences were found between the groups at baseline (*p* > 0.05), except for maternal age at birth (*p* < 0.05). No adverse effects or injuries were reported in either group.

**Table 1:** Sociodemographic and baseline demographic characteristics of intervention groups.

Variables		HEP		TT		Total		p
		*n*	%	*n*	%	*n*	%	
Sex	Female	6	40.0	5	35.7	11	37.9	0.558^a^
	Male	9	60.0	9	64.3	18	62.1	
Mother's educational level	Secondary school	1	6.7	1	7.1	2	6.9	0.574^a^
	High school	2	13.3	2	14.3	4	13.8	
	Bachelor degree	8	53.3	10	71.4	18	62.1	
	MSc, PhD	4	26.7	1	7.1	5	17.2	
Father's educational level	Secondary school	0	0	0	0	0	0	0.442^a^
	High school	4	26.7	2	14.3	6	20.7	
	Bachelor degree	11	73.3	11	78.6	22	75.9	
	MSc, PhD	0	0.0	1	7.1	1	3.4	
Multiple births	Single	13	86.7	8	57.1	21	72.4	0.086^a^
	Twins	2	13.3	6	42.9	8	27.6	
Family income	1–2 minimum wage	5	33.3	3	21.4	8	27.6	0.749^a^
	3–4 minimum wage	6	40.0	6	42.9	12	41,4	
	+5 minimum wage	4	26.7	5	35.7	9	31,0	
		Mean	SD	Mean	SD	t	df	p
Maternal age at infant's birth (years)		31.27	3.80	34.36	3.56	−2.25	27	**0.033^b^**
Age of father at infant's birth (years)		32.93	3.55	33.50	3.77	−0.41	27	0.681^b^
Birth gestational age (weeks)		28.93	2.81	29.79	3.53	−0.72	27	0.481^b^
Corrected age at baseline (weeks)		25.13	7.99	24.14	8.63	0.32	27	0.751^b^
Length of stay in the NICU (days)		51.60	19.51	57.71	28.08	−0.68	27	0.506^b^
Birth weight (gr)		1,429.27	336.36	1,411.14	535.05	0.11	27	0.913^b^

Bold values indicate statistical significance (*p* < 0.05).

df, degrees of freedom.

^a^ Chi square test.

^b^ Independent sample *t* test.

### 3.2 Primary outcome measures

#### 3.2.1 The peabody developmental motor scales-2 (PDMS-2)

A significant time effect was observed across all subtests and total scores of the PDMS-2, indicating improvements in motor skill levels over time in both groups (Table 2).

**Table 2:** The PDMS-2, TSFI scores of infants, and BAI scores of their parents.

Outcomes	HEP (*n* = 15)			TT (*n* = 14)			Baseline	Time effect			Time × Group		
	BI Mean (SD)	AI Mean (SD)	Estimate of Effect (95% CI)	BI Mean (SD)	AI Mean (SD)	Estimate of Effect (95% CI)	p*	*F*	p**	*P_η_* ^2^	*F*	p**	*P*_η_^2^
PDMS-2													
GMQ	23.47 (3.68)	30.47 (3.62)	−8.86/−5.14	23.64 (4.32)	27.29 (1.93)	−6.06/−1.21	0.907	57.194	**<0.001**	0.679	5.691	**0.024**	0.174
Reflexes	8.4 (1.4)	10.53 (0.99)	−2.33/−1.36	8.35 (1.44)	9.71 (0.46)	−2.37/−0.79	0.936	50.739	**<0.001**	0.653	2.509	0.125	0.085
Stationary	7.93 (1.43)	10.33 (1.23)	−3.08/−1.71	7.86 (2.10)	9.36 (0.84)	−2.77/−0.22	0.910	34.945	**<0.001**	0.564	1.861	0.184	0.064
Locomotion	7 (2.29)	9.87 (1.64)	−4.41/−1.31	7.43 (1.34)	8.14 (1.16)	−1.48/−1.31	0.549	18.909	**<0.001**	0.412	6.831	**0.014**	0.202
FMQ	16.93 (1.71)	21.93 (2.63)	−6.68/−3.31	16.86 (1.83)	17.93 (3.14)	−3.02/−3.31	0.909	25.839	**<0.001**	0.489	10.818	**0.003**	0.286
Grasping	7.6 (0.82)	11.2 (1.85)	−4.64/−2.55	7.57 (1.08)	8.07 (0.82)	−0.99/−0.006	0.937	55.612	**<0.001**	0.673	31.792	**0.000**	0.541
VMI	9.33 (1.04)	10.93 (1.38)	−2.31/−0.88	9.21 (1.05)	9.14 (0.66)	−0.45/−0.88	0.762	13.231	**≤0.001**	0.329	15.819	**0.000**	0.369
TMQ	40.40 (4.77)	52.33 (5.3)	−14.33/−9.53	40.50 (5.51)	44.50 (2.90)	−6.86/−1.13	0.959	85.149	**<0.001**	0.759	21.109	**0.000**	0.439
TSFI													
Total	30.53 (4.03)	45.27 (2.52)	−17.40/−12.05	28.93 (4.21)	38.50 (3.13)	−11.42/−7.72	0.304	250.881	**<0.001**	0.903	11.316	**0.002**	0.295
RTDP	9 (1.69)	9.93 (0.25)	−1.85/−0.01	8.71 (1.13)	9.71 (0.46)	−1.55/−0.44	0.601	14.348	**≤0.001**	0.347	0.017	0.897	0.001
AMF	5.13 (2.13)	12.80 (1.56)	−8.57/−6.76	4.36 (2.79)	9.29 (2.33)	−6.25/−3.59	0.406	291.891	**<0.001**	0.915	13.794	**0.001**	0.338
VTI	6.53 (1.80)	9.73 (0.45)	−4.18/−2.21	6.71 (1.59)	8.07 (0.99)	−2.43/−0.28	0.778	45.310	**<0.001**	0.627	7.410	**0.011**	0.215
OMC	1.53 (0.51)	2 (0)	−0.75/−0.18	1.43 (0.75)	1.93 (0.26)	−0.87/−0.12	0.665	19.787	**<0.001**	0.423	0.024	0.879	0.001
RVS	8.07 (1.98)	11 (1.46)	−4.05/−1.81	7.71 (1.59)	9.36 (1.27)	−2.47/−0.80	0.603	48.658	**<0.001**	0.643	3.869	0.60	0.125
BAI	15.20 (9.41)	13.07 (7.75)	0.54/3.72	12.57 (6.65)	12.14 (7.00)	−0.46/3.72	0.396	8.72	**0.006**	0.244	3.861	0.060	0.125

Bold values indicate statistical significance (*p* < 0.05).

*n*, sample size; HEP, homeostasis-enrichment-plasticity; TT, traditional treatment; ES, effect sizes; CI, confidence interval; BI, before intervention; AI, after intervention; SD, standard deviation; PDMS, Peabody Developmental Motor Scales; GMQ, gross motor quotient; FMQ, fine motor quotient; TMQ, total motor quotient; VMI, visual-motor integration; TSFI, test of sensory functions in infants; RTDP, reactivity to tactile deep pressure; AMF, adaptive motor function; VTI, visual tactile integration; OMC, ocular motor control; RVS, reactivity to vestibular stimulation; BAI, the Beck Anxiety Inventory; p*Independent sample *T*-Test; *p* < 0,05; Before Intervention/Baseline; p**Repeated Measures Analysis of Variance-rANOVA; *p* < 0,05; *P*_η_^2^ Partial Eta Squared.

Significant time × group interactions were identified in Grasping (*F* = 31.792, *p* < 0.001, *P_η_*^2^ = 0.541), Locomotion (*F* = 6.831, *p* = 0.014, *P_η_*^2^ = 0.202), Visual-Motor Integration (*F* = 15.819, *p* < 0.001, *P_η_*^2^ = 0.369), Fine Motor Quotient (*F* = 10.818, *p* = 0.003, *P_η_*^2^ = 0.286), Gross Motor Quotient (*F* = 5.691, *p* = 0.024, *P_η_*^2^ = 0.174), and Total Motor Quotient (*F* = 21.109, *p* < 0.001, *P_η_*^2^ = 0.439) in favor of the HEP Approach.

#### 3.2.2 Test of sensory functions in infants (TSFI)

Significant time effects were found across all TSFI subtests, reflecting improvements in sensory functions over time in both groups (Table 2).

Time × group interactions were significant for Adaptive Motor Function (*F* = 13.794, *p* = 0.001, *P_η_*^2^ = 0.338), Visual-Tactile Integration (*F* = 7.410, *p* = 0.011, *P_η_*^2^ = 0.215), and Total score (*F* = 11.316, *p* = 0.002, *P_η_*^2^ = 0.295), demonstrating more significant improvements in the HEP Approach group.

### 3.3 Secondary outcome measures

#### 3.3.1 Beck anxiety inventory (BAI)

A significant time effect was observed in parental anxiety levels measured by the BAI, indicating reductions over time in both groups (*F* = 8.72, *p* = 0.006, *P_η_*^2^ = 0.244) (Table 2). However, the time × group interaction was not significant (*p* > 0.05), suggesting similar reductions in anxiety across the HEP Approach and TT groups.

## 4 Discussion

This study investigated the effects of the HEP Approach on motor and sensory development in preterm infants and parental anxiety relative to TT. Data from 29 of the 32 randomized infants were evaluated. The average gestational age suggested a cohort of preterm infants at increased developmental risk (38, 39). Except for maternal age, the groups were sociodemographically analogous. Following a 12-week period, both therapies enhanced infant outcomes and alleviated parental anxiety, with the HEP Approach producing markedly superior improvements in motor and sensory scores.

At baseline, it was observed that the infants' pre-treatment mean scores for stationary, locomotion, and grasping were below the normative data, indicating delayed motor skill development compared to full-term infants (38, 40). In motor skills, infants demonstrated statistically significant improvement in all subtests and PDMS-2 quotient scores, with a large effect size, over time.

The TT intervention often encompasses activities aimed at enhancing righting and balance reactions, as well as facilitating trunk control. The literature indicates that TT is expected to enhance motor development, particularly reflexes and stationary skills (9, 41). Consistent with previous studies, TT was found to effectively improve postural control and balance over time (41, 42). Moreover, substantial changes in fine motor (grasping and visual-motor skills) scores indicate that improved postural control promotes manipulative skills over time, consistent with previous research findings (43).

For the HEP Approach group, it was hypothesized that the significant improvements in the gross, fine, and total motor skills of preterm infants could be attributed to the collaboration between the therapist and the family. This collaboration ensures that the child receives continuous, individualized environmental stimulation, encouraging active exploration and participation. This hypothesis is supported by previous studies demonstrating the positive outcomes of the HEP Approach on motor development in infants (22–24). Additionally, prior case reports have shown that motor development in infants with cerebral palsy improved following HEP Approach intervention (23, 24). Furthermore, a recent feasibility study revealed that the HEP Approach improved motor development in premature infants at developmental risk (24).

Upon comparing the groups, the HEP Approach was found to be superior to the TT intervention in improving fine motor, gross motor, total motor scores, and all other PDMS-2 subscales, except for reflexes and stationary. This similarity in reflex and stationary subscales may be attributed to the distinct focus of each intervention. The TT intervention is specifically designed to improve reflex responses, balance reactions, and postural control, which probably explains its direct effectiveness in these areas. In contrast, the HEP Approach indirectly creates opportunities that facilitate reflex development and stationary skills through family-led continuous, individualized environmental adaptations and activity choices. This suggests that the HEP Approach may serve as an effective alternative to TT for supporting reflex and stationary motor skills. However, for other subscales, including grasping, locomotion, and visual-motor integration, the HEP Approach demonstrated more significant improvements.

The reason for this superiority may lie in the more targeted, dynamic, and personalized nature of the HEP Approach. Unlike TT, which focuses on general postural control and balance, the HEP Approach provides continuous and individualized environmental stimulation tailored to the needs of each infant, thus fostering more effective motor development (22–24). Numerous studies support the importance of the environment, objects, and interactions with people in promoting motor development (44–50). For instance, Hewitt et al. demonstrated the positive effects of environmental modification and appropriate object use on tolerance to the prone position, which directly impacts locomotion development (51). Other studies have also found that placing infants on inclined surfaces with a preferred object increases head lifting in the prone position, promoting gross motor development (52–54). Similarly, Morgan et al. and Dusing et al. observed that providing appropriate toys, organizing the environment, and supporting infant movements positively enhanced motor skills (55).

In the HEP Approach, it is crucial to facilitate the utilization of infants' strongest sensory systems (24). Therefore, the objective is to position infants with sufficient visual perception in a vertical position (either sitting or standing), enabling them to develop improved awareness of their bodies and environment (22–24). This, in turn, enhances their ability to use their bodies and engage with their environment. Furthermore, numerous studies have shown that vertical positions are linked to hand-eye coordination, manipulation skills, joint attention, reaching, and grasping abilities (22, 56–60). Kretch et al. investigated the relationship between sitting and infants' exploration and interactions in typically developing infants aged 4–7 months and infants with motor development delays aged 7–16 months. Findings demonstrated that supported infants in both groups interacted less with their caregivers (by looking away more) and objects (by grasping and touching them less) than unsupported infants during free play. At the same time, caregivers who had to support their infants with both hands were also less able to use objects for interaction (57). Harbourne et al. also found that the fine motor skills of infants with motor delays improved after 12 weeks of the START-Play intervention. The researchers suggested that this improvement in fine motor skills was related to the infants' early vertical positioning (61).

According to the evaluation of sensory functions with the TSFI, the infants' baseline AMF, RVS, and Total Scores in this study in both groups were found to be at risk or deficient depending on their age. Similarly, Cabral et al. found that preterm infants had lower RTDP and Total Scores than term infants in sensory functions evaluated with TSFI (62). These results suggest that early interventions should focus on both sensory functions and motor skills in preterm infants.

Infants showed statistically significant improvement in all domains of the TSFI (RTDP, AMF, VTI, OMC, RVS, and Total Scores), with large effect sizes over time. Improvements in sensory functions within the TT intervention over time are thought to arise from vestibular, proprioceptive, and tactile sensory inputs produced during therapeutic handling activities. Similarly, Habik-Tatarowska found that a four-month intervention, similar to the TT intervention in this study, had a positive effect on the sensory functions of infants aged 4–12 months (63). Thus, the significant improvements observed in TT may be attributed to these sensory experiences. In the HEP Approach, while no direct sensory activities were performed, the results were consistent with our hypothesis that sensory improvements could result from the infant's increased capacity for movement and active exploration (22–24). According to the Perception-Action Theory, sensory functions and motor skills are intrinsically linked, with perception informing motor activity and motor development enhancing sensory experiences (44, 45). Research by Adolph and Franchak supports this, noting that motor behavior enriches perceptual experiences and offers feedback that influences subsequent actions (64). Additionally, Adolph and Hoch suggested that motor development facilitates learning and exploration, promoting developmental changes across several domains (44). In this study, the HEP Approach likely improved sensory functions by enhancing the infants' movement abilities, which fostered sensory experiences and integration. These findings also align with sensory integration theory, which emphasizes the critical role of sensory inputs in development, including motor skills (65, 66).

When comparing the groups, the HEP Approach demonstrated significant improvement in sensory functions, including AMF, VTI, and Total Scores, with large effect sizes favoring the HEP Approach group. This is especially notable because sensory improvements were not a direct target of the intervention. It was hypothesized that these improvements were due to the increased sensory experiences provided by the infants’ enhanced movement capacity and active exploration. Despite the absence of direct sensory activities, the HEP Approach indirectly improved sensory functions through its focus on motor skills, highlighting the relationship between sensory and motor development (44, 64). These results align with studies emphasizing the importance of environmental stimulation in fostering both motor and sensory development in preterm infants (44–50).

The similarity in improvements over time for RTDP, OMC, and RVS subtests in both groups may be attributable to the characteristics of the therapies. Vestibular and tactile inputs from therapeutic handling activities commonly employed in TT intervention are believed to enhance these domains directly. The HEP Approach posits that an infant's spontaneous engagement with various sensory stimuli through active exploration indirectly fosters development in these domains. The HEP Approach, which yields comparable outcomes for advancing these areas, may serve as an alternative to the TT intervention.

These findings align with the broader literature, which underscores the relationship between motor development and sensory functions in preterm infants (63, 64). These results, particularly the improvement in sensory functions within the HEP Approach group, support previous studies suggesting that early interventions focusing on motor skills can also lead to significant gains in sensory functions (16, 22–24). The HEP Approach, by facilitating movement and active exploration, plays a key role in promoting both motor and sensory development, thus enhancing overall developmental outcomes for preterm infants.

Parents' adverse mental health, particularly anxiety, is a critical factor due to its adverse effects on infants' sensory functions, motor skills, and overall developmental progress (65, 67). Anxiety in caregivers can create a stressful environment that may hinder their ability to provide optimal care, which in turn can impact their child's growth and development. A systematic review and meta-analysis have indicated that preterm infant development is more closely associated with the anxiety levels of caregivers than their stress levels (68). Therefore, addressing the anxiety of caregivers from a holistic perspective is essential in early intervention studies to promote both the parent's well-being and the infant's development.

In this study, the mean anxiety level of the parents at baseline was mild, with a BAI score of 13.93. This indicates that, while the parents did experience anxiety, it was within the mild range at the beginning of the intervention. Given the challenges of caring for preterm infants, such as increased medical appointments and constant worry about their child's health, it is common for parents to experience heightened anxiety during the early stages. This finding aligns with the existing literature, where parents of preterm infants often report higher anxiety levels due to the increased uncertainty and challenges associated with caring for their vulnerable infants (68, 69).

At the end of the intervention, there was a significant reduction in anxiety scores for all participants, with large effect sizes observed. This indicates that both the HEP Approach and TT interventions were effective in reducing parental anxiety. However, when comparing the two groups, no significant interaction between group and time was found. This suggests that while both interventions resulted in significant reductions in caregiver anxiety, there was no marked difference in the extent of improvement between the two groups.

These findings suggest that early interventions for preterm infants can significantly reduce caregiver anxiety, even when baseline anxiety levels are mild. Both the HEP Approach and TT interventions were similarly effective in addressing caregiver anxiety. This supports the broader literature on family-centered interventions, which emphasizes the importance of caregiver mental health in improving both parent and infant outcomes (65, 67, 70). Future research could explore additional factors to enhance the efficacy of these interventions and further improve caregiver well-being.

The HEP Approach closely aligns with the fundamental principles of occupational therapy, particularly in its emphasis on promoting meaningful participation, enhancing functional outcomes, and utilizing activity-based and family-centered practices. From an occupational therapy standpoint, the HEP Approach transcends mere skill learning by facilitating infants' engagement in meaningful activities within enriched and ecologically relevant environments. The HEP Approach's organized yet adaptable framework enables caregivers to modify the physical and social environment to facilitate the infant's active participation, self-regulation, and sensory-motor development. This reflects the emphasis of occupational therapy on enabling individuals to participate in daily routines and meaningful occupations across settings. Moreover, the incorporation of parental coaching, environmental adjustments, and infant-led exploration within the HEP Approach aligns with the PEO model commonly utilized in occupational therapy. These findings support the view that the HEP Approach is not only effective in improving developmental outcomes but also embodies occupational therapy principles in both philosophy and practice.

This study offers valuable insights into the effectiveness of the HEP Approach in preterm infants; however, several limitations exist. The sample consisted of preterm infants with a mean gestational age of 28.9 weeks and a mean current age of 6 months, as well as parents with a relatively high level of education. Additionally, infants with grade III/IV intraventricular hemorrhage or significant sensory impairments were excluded to maintain methodological rigor and a relatively homogeneous sample for this study. Although referrals came from three different hospitals, all participants were from a single geographical region and received assessment and intervention at the same center, which may reflect limited cultural and healthcare variation. These clinical and sociodemographic characteristics may limit the generalizability of the results to more vulnerable preterm infants with diverse backgrounds. Furthermore, while significant improvements were observed, the intervention was limited to a 12-week period and lacked long-term follow-up, which prevents understanding whether these benefits are sustained over time. However, evidence from a single-case study of an infant with hemiparetic cerebral palsy and twin anemia polycythemia sequence reported that developmental gains were sustained or enhanced at a 4-month follow-up, particularly in sensory and motor outcomes (23). Although these findings provide preliminary support for the potential sustainability of HEP Approach intervention effects, further controlled studies with larger sample sizes and longer-term follow-up are necessary. A potential limitation was that, although the researchers did not provide the intervention for the control group, it was conducted by experienced NDT/Bobath trained therapists, and detailed records of the interventions were kept. This ensured consistency in the application and mitigated potential bias, preventing it from being a limitation in the study. In addition, the study did not assess alterations in infants' functional performance or their engagement in daily activities, thus constraining the interpretation of how developmental achievements manifest in everyday contexts. Although the HEP Approach led to significant changes, it was not explicitly designed to target sensory enrichment; therefore, the observed effects may be due to increased movement and exploration rather than the sensory components alone. Nonetheless, this study represents the first RCT to explore the HEP Approach in preterm infants, providing valuable preliminary data. Future research should adopt broader inclusion criteria, including preterm infants with more complex clinical profiles such as high-grade intraventricular hemorrhage or significant sensory impairments, implement an extended follow-up period, and utilize multi-center designs with socioeconomically and culturally diverse samples to enhance the generalizability and validity of findings. Moreover, ecologically valid tools such as Goal Attainment Scaling (GAS), the Canadian Occupational Performance Measure (COPM), or the Young Children's Participation and Environment Measure (YC-PEM) should be considered to assess real-world functional outcomes and participation. In addition, future studies could investigate the feasibility of implementing the HEP Approach in home-based, hybrid or telehealth formats, which may further increase accessibility and applicability across diverse settings.

## 5 Conclusions

In conclusion, after 12 weeks of early interventions, significant improvements in motor skills and sensory functions were observed in preterm infants, along with a reduction in caregiver anxiety. The HEP Approach led to superior improvements in fine and gross motor skills, as well as certain sensory functions, compared to the TT intervention. Both the HEP Approach and TT interventions were similarly effective in reducing caregiver anxiety.

These findings underscore the significance of early interventions in promoting both infant development and caregiver well-being. The HEP Approach, in particular, could be integrated into standard care practices for preterm infants, offering a compelling alternative intervention for improving developmental outcomes. This study suggests that the HEP Approach may be a useful EI model for physical and occupational therapists to promote developmental gains and ultimately functional performance in preterm infants. The integration of numerous occupational therapy theories makes this approach uniquely suited to occupational therapists. Future research should focus on examining the long-term effects, sustainability, and broader applicability of these interventions across diverse populations and clinical settings to refine care strategies.

## Data Availability

The original contributions presented in the study are included in the article/Supplementary Material, further inquiries can be directed to the corresponding author.
