# Supplementary material for: Effects of the HEP® (Homeostasis–Enrichment–Plasticity) Approach in preterm infants with increased developmental risk: a randomized controlled study
Source: Front Pediatr. 2025 Sep 25;13:1606490. doi: 10.3389/fped.2025.1606490 (PMC12509065; doi:10.3389/fped.2025.1606490)
Supplement: Supplementary file 1 [file Table1.docx]

Supplementary Material

**Table S1. Comparison of the HEP Approach with START-Play and GAME Interventions.**

|  | START-Play (1-4) | | GAME (5-7) | HEP (8-12) |
| --- | --- | --- | --- | --- |
| Delivery mode | - Delivered to parent/caregiver and child - 40–60 min - Twice weekly | | - Delivered to parent/caregiver and child - 60–90 min - Initially weekly, then frequency adjusted with the family (5,6) *or at* least fortnightly (7) | - Delivered to parent/caregiver and child - 45–60 min - Weekly |
| Setting | - Home-based (1-3) *or,* depending on caregiver preference, home-based or clinical-based (4) | | - Home-based | - Clinic-based intervention and parent coaching with parallel parent-implemented home-based activities with therapist monitoring of infants’ natural environments via online follow-up. |
| Parent role | - Active collaborator | | - Active collaborator | - Active collaborator |
| Theoretical models | - Dynamic Systems Theory - Embodied Cognition Framework | | - Dynamic Systems Theory - Ecological framework - Motor learning principles - Family-centred practice - Environmental enrichment strategies | - Dynamic Systems Theory - Gibson’s Ecological Perception Theory - Neuronal Group Selection Theory - Perception-Action Theory - Sensory Integration Theory - Person–Environment–Occupation (PEO) Model |
| Scope of enrichment principles | Homeostasis | - Not reported in the intervention details. | - Addressed directly - Therapist supports the family with feeding and positioning strategies (e.g., anti-reflux), optimizing sleep–wake routines, and approaches to assist the infant’s state regulation. | - Addressed directly - Therapist supports families in monitoring and addressing both their own and the infant’s well-being (sleep, feeding, overall health, regulation) and encourages balanced routines and self-care. |
|  | Safety | - No explicit reference to social or physical safety. | - Addressed indirectly - Manual support is provided only when necessary for safety or to give the infant the “idea” of the movement, then promptly removed once self-initiation is observed. | - Addressed directly - Parents are guided to create secure contexts, provide verbal/emotional reassurance, introduce new situations, and use simple environmental adjustments (e.g., rubber tube) for safe exploration. |
|  | Sensory Experiences | - Addressed indirectly - Infants’ exploration of objects, body–object affordances, and play in natural environments supports their sensory experiences. | - Addressed indirectly - Infants' practice of reaching for and grasping various objects supports their sensory experiences. | - Addressed directly - Caregivers are supported to provide active, meaningful sensory experiences tailored to infant strengths (e.g., upright visual exploration with bouncers/baskets) and to examine environmental and object sensory features to support the infant’s sensory-motor challenges and to provide a just-right interaction challenge. |
|  | Spatial | - Addressed indirectly - Using variable sitting positions and taking advantage of environmental opportunities supports the expansion of the infant’s exploration area. | - Addressed indirectly - Using sitting positions and encouraging families to leave the house supports the expansion of the infant’s exploration area. | - Addressed directly - Families are guided on how room features, equipment, and objects shape exploration, with recommended spatial modifications (e.g., larger rooms, stair access, textured cushions) and expansion of explored spaces. |
|  | Novelty | - Addressed indirectly - Variable child-initiated movements, integration of cognitive constructs with motor tasks, and problem-solving activities designed with parents create opportunities for novelty that enhance learning and exploration. | - Addressed directly - Variable practice, including the use of different objects for reaching and grasping and environmental arrangements, introduces novelty that increases task complexity and encourages exploration. | - Addressed directly - Families encourage babies to explore movement in novel environments with baby walkers for increased age-appropriate mobility and enrich their experiences by making small changes to their routines and environment, such as rearranging furniture, changing song rhythms, and altering feeding positions. |
|  | Challenge | - Addressed directly - Task difficulty is increased incrementally, with parents collaborating to provide “just right” challenges that blend motor and cognitive skills. | - Addressed directly - Tasks and environmental conditions are progressively modified to match the infant’s improving performance and to encourage problem-solving. | - Addressed directly - Parents are guided on how to incorporate stimulating challenges into daily routines. Challenges are aligned with the child’s zone of proximal development to foster integration across physiological systems, praxis abilities, sensory processing and enhance overall development. Activities are adapted to the infant’s abilities for a just-right challenge (e.g., pillows for support, uneven surfaces, object-hiding games). |
|  | Enjoyment | - Addressed indirectly - Play-based activities, object-oriented exploration, and caregiver–infant interactions support the idea of enjoyment. | - Addressed indirectly - Play preferences, family involvement, and self-directed activities support the idea that practice remains enjoyable and engaging for the infant. | - Addressed directly - Parents are guided to create social and physical environments that enable enjoyable exploration with favorite toys and supportive interactions. The importance of enjoyment is emphasized as essential for motivating learning and encouraging repetition. |
|  | Continuous Engagement | - Addressed directly - Intensive daily activities, consistent visits, and repeated opportunities for play and problem-solving are embedded within the infant’s daily routines in the natural environment. | - Addressed directly - Active practice opportunities are embedded within the infant’s daily routines, with parents sustaining participation through a structured, goal-focused home program. | - Addressed directly - Parents are empowered to develop critical thinking and problem-solving skills, creating opportunities for independent exploration and learning within their infants’ daily routines; the use of easily accessible objects such as baskets and books is encouraged to sustain active exploration. |
|  | Social | - Addressed directly - Caregiver social support, joint attention, and parent–infant interactions are used to scaffold skill development. | - Addressed directly - Parents, siblings, and extended family are involved in therapy and home programs to promote interaction, family acceptance, and natural opportunities for varied social engagement. | - Addressed directly - Families are coached on how social relationships support learning and development. They are guided in using effective communication strategies and encouraged to provide exposure to varied social settings to enrich the child’s experiences. |
|  | Active Engagement and Exploration | - Addressed directly - Self-initiated, goal-directed movements are promoted through variable child-initiated play and problem-solving supported by environmental opportunities. | - Addressed directly - Self-generated, goal-directed motor activity is fostered by encouraging voluntary movement attempts and providing scaffolded tasks, with environmental and object opportunities supporting exploration. | - Addressed directly - The role of people, environments, and resources in fostering active exploration is emphasized. Families are guided to provide sufficient time and safe settings—for example, removing carpets for walker use or encouraging object play in sitting positions. |

Note. Dark shaded cells represent areas where all three interventions address the intervention component.

Copyright © 2025, Balikci, Sirma, & May-Benson. Reproduced from The HEP® (Homeostasis-Enrichment-Plasticity) Approach Manual. Used with permission.

**References**

1. Harbourne RT, Dusing SC, Lobo MA, Westcott-McCoy S, Bovaird J, Sheridan S, Galloway JC, Chang HJ, Hsu LY, Koziol N, Marcinowski EC, Babik I. Sitting together and reaching to play (START-Play): protocol for a multisite randomized controlled efficacy trial on intervention for infants with neuromotor disorders. *Phys Ther*. 2018;98(6):494–502.
2. Harbourne RT, Dusing SC, Lobo MA, McCoy SW, Koziol NA, Hsu LY, Willett S, Marcinowski EC, Babik I, Cunha AB, An M, Chang HJ, Bovaird JA, Sheridan SM. START-Play physical therapy intervention impacts motor and cognitive outcomes in infants with neuromotor disorders: a multisite randomized clinical trial. *Phys Ther*. 2021;101(2):1–11.
3. Koziol NA, Kretch KS, Harbourne RT, Lobo MA, McCoy SW, Molinini R, Hsu LY, Babik I, Cunha AB, Willett SL, Bovaird JA, Dusing SC. START-Play physical therapy intervention indirectly impacts cognition through changes in early motor-based problem-solving skills. *Pediatr Phys Ther*. 2023;35(3):293–302.
4. Koziol NA, Butera CD, Kretch KS, Harbourne RT, Lobo MA, McCoy SW, Hsu LY, Willett SL, Kane AE, Bovaird JA, Dusing SC. Effect of the START-Play physical therapy intervention on cognitive skills depends on caregiver-provided learning opportunities. *Phys Occup Ther Pediatr*. 2022;42(5):510–25.
5. Morgan C, Novak I, Dale RC, Badawi N. GAME (Goals-Activity-Motor Enrichment): protocol of a single blind randomised controlled trial of motor training, parent education and environmental enrichment for infants at high risk of cerebral palsy. *BMC Neurol*. 2014;14:203.
6. Morgan C, Novak I, Dale RC, Guzzetta A, Badawi N. Optimising motor learning in infants at high risk of cerebral palsy: a pilot study. *BMC Pediatr*. 2015;15:30.
7. Morgan C, Novak I, Dale RC, Guzzetta A, Badawi N. Single blind randomised controlled trial of GAME (Goals-Activity-Motor Enrichment) in infants at high risk of cerebral palsy. *Res Dev Disabil*. 2016;55:256–67.
8. Balıkcı A. Exploring effects of the HEP (Homeostasis-Enrichment-Plasticity) approach as a comprehensive therapy intervention for an infant with cerebral palsy: a case report. *J Child Sci*. 2022;12(1):e182–95.
9. Balıkcı A, May-Benson TA, Sırma GÇ, Ilbay G. HEP® (Homeostasis-Enrichment-Plasticity) approach changes sensory–motor development trajectory and improves parental goals: a single subject study of an infant with hemiparetic cerebral palsy and twin anemia polycythemia sequence (TAPS). *Children*. 2024;11(7):876.
10. Balıkcı A, May-Benson TA, Sırma GÇ, Kardas A, Demirbas D, Aracikul Balikci AF, Beaudry-Bellefeuille I. The Homeostasis-Enrichment-Plasticity (HEP®) approach for premature infants with developmental risks: a pre-post feasibility study. *J Clin Med*. 2024;13(18):5374.
11. Sirma, G.C. Investigation of the Effectiveness of Environmental Enrichment-Based I ̇ntervention in Preterm Infants. Master’s Thesis, I ̇stanbul University-Cerrahpasa Institute of Graduate Studies, Physiotherapy and Rehabilitation Programme, Istanbul, Turkiye, 2023.
12. Balikci A, Sirma GC, and May-Benson TA. *The HEP® (Homeostasis-Enrichment-Plasticity) Approach Manual.* Sense On, Istanbul, Türkiye; 2025. Unpublished manual.
