# Supplementary material for: Effects of the HEP® (Homeostasis–Enrichment–Plasticity) Approach in preterm infants with increased developmental risk: a randomized controlled study
Source: Front Pediatr. 2025 Sep 25;13:1606490. doi: 10.3389/fped.2025.1606490 (PMC12509065; doi:10.3389/fped.2025.1606490)
Supplement: Supplementary file 2 [file Table2.docx]

Supplementary Material

# Table S2. Examples of intervention practices according to the key elements of the HEP Approach.

| **Key Components of the HEP Approach** | **Parent/Caregiver Coaching Strategies** | **Activities and Strategies** |
| --- | --- | --- |
| **Homeostasis:**  The HEP Approach focuses on parental environmental (heat, noise, parental practices) and individual (health, sleep, eating habits, stress, regulation skills) factors influence homeostasis. Parents are guided on how their baby’s sleep, eating, and wake patterns impact development, and they receive individualized strategies to support regulation. | Therapists educate families on the importance of monitoring both their own and their baby's well-being while establishing routines for sleep and nourishment. Since parental well-being affects infant self-regulation, parents are encouraged to engage in self-care activities like socializing or walking. | Therapists provide regulation techniques such as pacifier use, baby walkers, and calming methods like physical contact, rhythmic rocking, and massage to help babies self-regulate effectively. |
| **Safety:**  For effective environmental interaction, an individual’s nervous system must perceive physical and emotional safety, which is essential for exploration, learning, and development. Therapists work with parents to adjust the environment based on the infant’s strengths and needs while also fostering parental confidence to support the baby’s growth. | Therapists educate parents on how a sense of security helps infants explore, gain new experiences, and develop sensory, motor, emotional, and cognitive skills. They emphasize that babies rely on their parents for safety, so parents must feel secure and be responsive. | Therapists encourage parents to provide verbal and emotional reassurance to support exploration when the baby feels insecure. For example, when meeting a new person, parents can introduce and explain the situation to the baby. Environmental adjustments, like using a rubber tube, help infants explore different surfaces, movements, and spaces safely. |
| **Sensory Experiences:**  Learning and development depend on varied sensory experiences and effective sensory processing, which allow individuals to explore and engage with their environment. Sensory systems help transmit accurate information to the nervous system, supporting exploration, engagement, learning, and development. Therapists facilitate this process by arranging the environment and adjusting equipment to optimize sensory use. | Therapists inform caregivers about the importance of active and meaningful sensory experiences and emphasize how the baby’s strong sensory systems support exploration, perception, and development. | Therapists design settings that promote active sensory exploration based on the infant’s strengths. For example, babies with strong visual perception benefit from activities in upright positions (standing, sitting) and the use of equipment like baby bouncers or baskets, enabling them to explore their body, surroundings, and objects through sight. |
| **Spatial:**  The physical characteristics of space—including room size, support surfaces, objects, and equipment—can significantly impact exploration, perception, learning, and development. Larger and well-structured spaces offer diverse opportunities for engagement and growth, especially when aligned with the child’s zone of proximal development. | Therapists inform families about how room features, equipment, and objects influence active exploration and learning and emphasize the importance of exposing infants to varied spatial environments. | To support spontaneous self-organization and active exploration, therapists recommend spatial modifications, such as larger rooms or stair access, and suggest appropriate equipment like cushions of different textures and heights or a baby walker. Caregivers are encouraged to expand the baby’s explored environment to enhance development. |
| **Novelty:**  Novel experiences—such as new items, toys, surfaces, and activities—support exploration, learning, and developmental change by providing sensory-motor, emotional, and cognitive stimulation. Therapists modify environments and tasks within the child’s zone of proximal development to create opportunities for new actions and experiences, working closely with parents to introduce novelty. | Therapists emphasize the importance of varied and new experiences for the baby’s development. They also learn about the family’s daily routines and suggest ways to incorporate new elements into them. | Therapists gradually adjust environments and activities to encourage exploration. For example, they introduce a baby walker for movement across different areas, rearrange furniture to promote mobility, and make small changes to routines, such as altering song rhythms or changing feeding positions, to enrich the infant’s experiences. |
| **Challenge:**  Challenges are essential for learning and development, encouraging more complex behaviors. However, tasks that are too easy or too difficult do not promote effective learning. Challenges should be aligned with the child's zone of proximal development to support integration across physiological systems and enhance overall development. | Therapists guide parents on incorporating appropriate challenges into daily routines, ensuring they are achievable yet stimulating for the infant. | Therapists modify environments and activities based on the child's abilities to encourage progress. For example, they:  -Use pillows for lateral support in a laundry box to help with mobility exploration.  -Introduce uneven surfaces for a crawling baby to develop alternative movement strategies.  -Suggest object-hiding games for babies who can already uncover a parent’s face, expanding their problem-solving skills. |
| **Enjoyment:**  Meaningful, intentional, and enjoyable activities enhance learning and development by encouraging repetition, leading to neurophysiological changes and adaptations. Therapists work with families to design environments and activities that align with the child’s abilities and create positive experiences. | Therapists guide parents in organizing social and physical environments to foster pleasurable exploration, incorporating optimal gestural responses and favorite toys into activities. | Therapists emphasize the role of enjoyment in learning, helping parents motivate their child to explore movements, surroundings, and objects through structured, enjoyable experiences. |
| **Continuous Engagement:**  Repetition is key to learning and developmental change, requiring regular opportunities for active engagement with the environment. Strategies must be easily implementable in different settings and times to ensure consistent exploration. | Therapists emphasize the need for continuous active exploration and highlight the family’s role in supporting this process. | Therapists help families identify growth opportunities that align with their baby’s needs and daily routines. By encouraging critical thinking and problem-solving, parents learn to create appropriate opportunities for independent exploration and learning. |
| **Social:**  The social environment, including adults and peers, plays a crucial role in learning and development by providing opportunities for interaction, exploration, and engagement. A varied and supportive social setting fosters growth within the child’s zone of proximal development. | Therapists educate parents and caregivers on the impact of social relationships on learning and development, emphasizing the value of diverse social experiences. | Therapists encourage parents to reflect on effective relationship-building strategies by asking insightful questions like, “What type of feedback motivates your child?” or “How do you support their learning efforts?”. This helps parents identify optimal communication methods, such as balancing verbal and nonverbal cues or involving the child in family interactions. Therapists also provide tailored strategies and encourage parents to expose the child to varied social settings for enriched development. |
|  |  |  |
| **Active Engagement and Exploration:**  Active exploration enhances perception, learning, and development by encouraging the child to interact with their physical and social environment. Therapists help design tailored environments that support exploration and participation. | Therapists highlight the role of people, surroundings, and available resources in promoting active exploration. | Therapists emphasize allocating enough time for each child’s unique exploration process. They guide parents in selecting suitable environments, tools, and communication methods to enhance engagement. For example:  -Removing carpets to improve mobility with a baby walker.  -Encouraging object exploration in a sitting position rather than lying down. |
