# Supplementary material for: Effects of the HEP® (Homeostasis–Enrichment–Plasticity) Approach in preterm infants with increased developmental risk: a randomized controlled study
Source: Front Pediatr. 2025 Sep 25;13:1606490. doi: 10.3389/fped.2025.1606490 (PMC12509065; doi:10.3389/fped.2025.1606490)
Supplement: Supplementary file 3 [file Table3.docx]

Supplementary Material

# Table S3. Practice examples and goals of TT intervention.

| Practice Examples | Goals |
| --- | --- |
| Facilitation of the transition from supine to sitting position | Activation of the abdominal oblique muscles  Upper limb weight transfer  Providing independent transition |
| Asymmetric/symmetric rotation in supine position with hands reaching to knees and/or feet | Elongation of the spinal extensors, activation of the neck and trunk flexors, hip and knee flexion.  Increasing sensory awareness of the right and left side of the body  Body exploration with hands and visual field  Ensuring independent rotation |
| Reaching in the prone position | Elongation of the rectus abdominus and hip flexors  Head, neck and hip extension  Stimulation of visual, tactile, proprioceptive and vestibular systems  Preparation for prone position activities |
| Prone-extension in prone position on the pilates ball | Head and trunk extension  Symmetrical hip and knee extension  Protective extension forward of the upper limb (to protect the baby when falling)  Vestibular and proprioceptive stimulation |
| Transferring weight while sitting on a pilates ball | Weight transfer to the front for activation of the trunk extensor muscles  Back weight transfer for activation of the trunk flexor muscles  Elongation with eccentric muscle activation on the weight transfer side, lateral weight transfer for concentric muscle activation on the non-transfer side |
| Diagonal weight transfer in sitting position on the leg | Elongation of the hip adductors  Lower limb dissociation  Hip joint mobilisation  Increasing spinal rotation to improve breathing |
| Transition from long sitting to crawling position | Elongation of the quadriceps muscle, hip internal rotator and adductor muscles  Shoulder flexion with trunk extension  Upper limb weight transfer  Transition from sitting to three/four point position |
| Transition from prone position to standing position | Transition from prone position to 4 foot position  Transition from 4 foot position to kneeling position  Transition from kneeling position to knight position and standing position  Elongation of the quadriceps and hip flexors  Activation of hip extensors and abductors  Hip extension with knee flexion  Trunk extension with hip extension  Plantar flexion - dorsi flexion transition  Weight shift |
