# Supplementary material for: Effects of the HEP® (Homeostasis–Enrichment–Plasticity) Approach in preterm infants with increased developmental risk: a randomized controlled study
Source: Front Pediatr. 2025 Sep 25;13:1606490. doi: 10.3389/fped.2025.1606490 (PMC12509065; doi:10.3389/fped.2025.1606490)
Supplement: Supplementary file 4 [file Table4.docx]

Supplementary Material

# Table S4. Timeline-Based Comparison of HEP Approach and Traditional Treatment.

|  | **HEP Approach** | | **Traditional Treatment** | |
| --- | --- | --- | --- | --- |
|  | Description | Examples | Description | Examples |
| **Weeks 1–4** | Focus on strategies to promote homeostasis and self-regulation, addressing basic needs such as sleep-wake cycles, feeding, safety, and emotional regulation. | Modeling co-regulatory behaviors (e.g., rhythmic rocking, pacifier use, soft touch). | Hands-on facilitation of key motor transitions (supine ↔ sitting), targeting activation of abdominal, trunk, and cervical musculature. | Manual support to promote midline orientation and segmental rolling. |
| **Weeks 2–6** | Support families in organizing the physical and social environment to promote active exploration, tailored to the infant’s and family’s individual characteristics. | Reorganizing the room to promote exploration; use of visually engaging upright positions like laundry baskets or walkers. | Guided humeral and forearm movement to improve reaching patterns, grasp reflex modulation, and supination for functional hand use. | Using thumbs and fingers to guide humeral adduction and elbow flexion while encouraging baby to reach mouth/face. |
| **Weeks 4–8** | Guide families in generalizing acquired skills across settings, using various objects, positions, and social partners. | Using familiar skills (e.g., reaching or crawling) across novel settings, toys, and social contexts. | Active weight-bearing through upper extremities in prone or supported sitting to build postural stability, proprioception, and strength. | Therapist facilitates weight-bearing and active pushing through hands on a firm surface or therapy ball in supported positions. |
| **Weeks 6–12** | Strengthen parental self-efficacy via reflective coaching on environmental adaptations, object/tool use, task variation, and caregiver-infant interaction quality. | Prompting caregivers with reflective questions such as “What motivated your baby more today?” to support environmental tuning. | Focus on dynamic postural control through crawling, kneeling, and transitions to improve proximal stability. | Crawling drills, quadruped-to-kneeling transitions with trunk activation. |
